# Generation of an Immortalized Human Adipose-Derived Mesenchymal Stromal Cell Line Suitable for Wound Healing Therapy

**DOI:** 10.3390/ijms23168925

**Published:** 2022-08-10

**Authors:** Daniela-Madalina Iacomi, Ana-Maria Rosca, Raluca Tutuianu, Tiberiu Paul Neagu, Vasile Pruna, Maya Simionescu, Irina Titorencu

**Affiliations:** 1 Cell and Tissue Engineering Laboratory, “Nicolae Simionescu” Institute of Cellular Biology and Pathology, 050568 Bucharest, Romania; 2 Clinical Department No. 11, “Carol Davila” University of Medicine and Pharmacy, 050474 Bucharest, Romania

**Keywords:** adipose-derived stromal cells, immortalization, hTERT, lentiviral transduction, secretome, wound healing

## Abstract

Adipose-derived mesenchymal stromal cells (ADSC) are a promising source for cellular therapy of chronic wounds. However, the limited life span during in vitro expansion impedes their extensive use in clinical applications and basic research. We hypothesize that by introduction of an ectopic expression of telomerase into ADSC, the cells’ lifespans could be significantly extended. To test this hypothesis, we aimed at engineering an immortalized human ADSC line using a lentiviral transduction with human telomerase (hTERT). ADSC were transduced with a third-generation lentiviral system and a hTERT codifying plasmid (pLV-hTERT-IRES-hygro). A population characterized by increased hTERT expression, extensive proliferative potential and remarkable (potent) multilineage differentiation capacity was selected. The properties for wound healing of this immortalized ADSC line were assessed after 17 passages. Their secretome induced the proliferation and migration of keratinocytes, dermal fibroblasts, and endothelial cells similarly to untransduced ADSC. Moreover, they sustained the complete re-epithelialization of a full thickness wound performed on a skin organotypic model. In summary, the engineered immortalized ADSC maintain the beneficial properties of parent cells and could represent a valuable and suitable tool for wound healing in particular, and for skin regenerative therapy in general.

## 1. Introduction

Skin wound healing is a highly coordinated process which reestablishes the integrity and the barrier function of this tissue. However, the progression of the healing process can be impaired by local factors (foreign bodies, tissue maceration, ischemia, infection) or systemic conditions (malnutrition, advanced age, diabetes, and renal disease), resulting in chronic wounds that fail to respond to regenerative stimuli [1]. It is likely that the incidence of chronic wounds will upsurge in the near future due to population aging and the prevalence of diabetes [2,3]. Furthermore, this pathological condition affects millions of patients worldwide every year, triggering a massive economic impact on healthcare systems [4]. Chronic wounds need advanced therapeutic approaches in order to enhance the healing process. Some of the recently developed strategies include negative-pressure wound therapy, hyperbaric oxygen therapy, biophysical approaches, local application of growth factors, acellular matrices, skin grafting, and cellular therapy [5]. Regarding the latter, mesenchymal stromal cells (MSC) are being considered as a powerful tool for the regenerative therapy of hard-to-heal wounds, due to their ease of harvest, low immunogenicity, and regenerative secretome, which has strong effects on the migration and proliferation of keratinocytes and dermal fibroblasts, as well as a pro-angiogenic effect [6,7]. MSC have been successfully isolated from a variety of tissues, such as bone marrow, adipose tissue, amniotic fluid and membrane, dental tissues, endometrium, limb bud, menstrual and peripheral blood, placenta and fetal membrane, salivary gland, skin and foreskin, sub-amniotic umbilical cord lining membrane, synovial fluid, and Wharton’s jelly [5]. From all of these locations, adipose tissue-derived MSC (ADSC) have been getting increased attention due to the ease of isolation and abundance [8]. Moreover, it has been shown that ADSC-derived secretome, which includes extracellular vesicles and soluble factors, such as cytokines and growth factors, have a notable impact on improving the healing process of cutaneous wounds [9,10,11]. Thus, keeping in mind the advantages of cell-free preparations over cellular products (such as avoiding the use of viable cells and diminishing the variability), developing a ready-to-use medicine based on ADSC-derived secretome for wound healing applications seems promising [12]. However, the development of such treatment requires the in vitro expansion of cells for a long period of time, which has the disadvantage that the biological material is quickly depleted and additional isolation of primary ADSC is needed, which could negatively impact the variability of the cell products. This depletion is due to the cell senescence occurring during in vitro propagation due to telomere shortening which leads to the decline of cell growth and modification of their properties [13,14]. This issue could be overcome by immortalization of cells, which can be attained by transduction of genes, such as the human telomerase reverse transcriptase (hTERT) [15,16,17]. By immortalization, a perpetual stem-cell source could be generated and the secretome harvesting protocol could be standardized to facilitate the development of an “off-the-shelf” medication, accessible for chronic wound treatment and for other types of skin injuries that require fast intervention, such as burns.

Here, we describe the generation of a single-cell-derived immortalized ADSC line generated by ectopic expression of hTERT using lentiviral gene transfer, which maintained the stem cell properties. Importantly, the secretome derived from the transduced cells exhibited a strong regenerative potential for wound healing as assessed on 2D cultured keratinocytes, human dermal fibroblasts (hDF) and endothelial cells (EC), and in a wounded 3D skin organotypic model. 

## 2. Results

### 2.1. Characterization of Human Adipose Derived Stromal Cells

**Figure 1:**
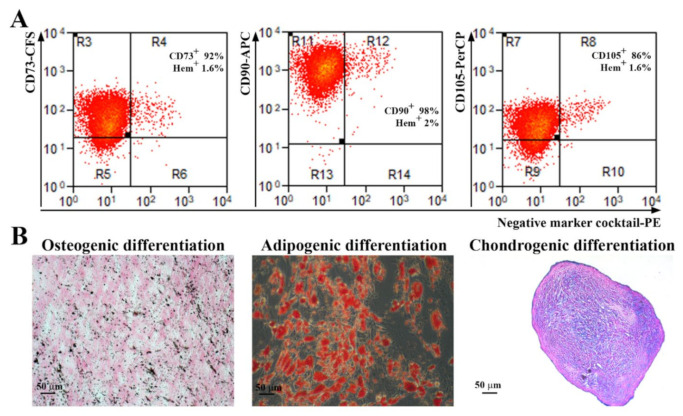
Characterization of human ADSC. (**A**) Representative flow cytometry histograms showing the expression of MSC-specific surface markers. It can be noticed that the cells were negative for the hematopoietic markers CD45, CD34, CD11b, CD79A, and HLA-DR, and positive for CD73, CD90, and CD105 (*n* = 3). (**B**) Analysis of the multipotent capacity of ADSC by their ability to generate osteocytes (left, von Kossa staining), adipocytes (middle, Oil Red O staining), and chondrocytes (right, Alcian blue staining) when incubated in specific conditions.

Abdominal subcutaneous adipose tissue-isolated ADSC were characterized according to the indications of the International Society for Cellular Therapy [18]. As revealed by flow cytometry analysis (Figure 1A), more than 90% of the cells were positive for CD73, CD90 and more than 80% for CD105 markers. Additionally, the cells were negative for the hematopoietic markers CD45, CD34, CD11b, CD79A, and HLA-DR (less than 2%). The gating strategy used for flow cytometry analysis and the negative controls for each antibody are shown in Supplemental Figure S1. Furthermore, when incubated in specific conditions, the cells were able to achieve trilineage differentiation (Figure 1B): osteogenic (revealed by calcium deposits in the extracellular matrix stained by von Kossa method), adipogenic (shown by Oil Red O staining of lipid droplet cytoplasmic accumulation), and chondrogenic (confirmed by staining of acid mucopolysaccharides of paraffin sections of the pellet).

### 2.2. Characterization of hTERT Transduced ADSC

**Figure 2:**
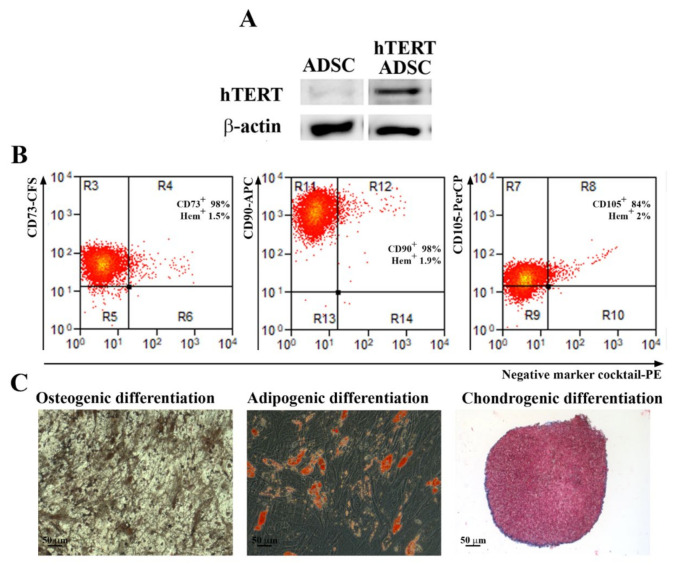
Analysis of hTERT expression, stem cell markers and differentiation potential of hTERT transduced ADSC. (**A**) Expression of hTERT before and after hTERT transduction as revealed by Western Blot technique. (**B**) Flow cytometry analysis showing that the expression of surface markers CD73, CD90 and CD105, required for mesenchymal stromal/stem cells definition, was not lost after lentiviral transduction up to the 31st passage (*n* = 3). (**C**) Evaluation of multilineage differentiation capacity of hTERT transduced ADSC. Note that, a short time after transduction (5th passage), the cells were able to properly differentiate into osteogenic and adipogenic lineages, and less into chondrocytes, since the Alcian Blue staining was hardly noticed.

Primary ADSC were transduced at passage 3 with a third-generation lentiviral system and a hTERT codifying plasmid (pLV-hTERT-IRES-hygro) as described above. After selection with hygromycin, a fibroblast-like population was obtained (Supplemental Figure S2) and characterized, in order to validate the induction of hTERT protein expression and to assess the preservation of the multipotent ability after transduction. As shown in Figure 2A, the hTERT protein was highly expressed in transduced cells, while the parental population had a very low expression (a barely visible band) as revealed by Western Blot assays. By flow cytometry it was determined that the transduced cells had an expression pattern of markers similar to the parental cells and consistent with multipotent MSC as defined by the International Society for Cellular Transplantation (Figure 2B). We found over 98% CD73 and CD90 positive cells, 84% CD105 positive cells, and less than 2% cells positive for hematopoietic markers. Moreover, this population kept its potential to differentiate into osteoblasts and adipocytes. However, the chondrogenic differentiation was somewhat impaired, as shown by the scarce staining by Alcian Blue of acid mucopolysaccharides in comparison to the parental ADSC that differentiated into chondrocytes. Therefore, to obtain a homogenous population with consistent stem cells characteristics, we proceeded to serial dilution cloning starting from the heterogenous population of transduced ADSC at passage 5.

### 2.3. Investigation of hTERT Expression, Proliferation Capacity and Senescence Marker β-Galactosidase of hTERT Transduced ADSC-Derived Sub-Populations

**Figure 3:**
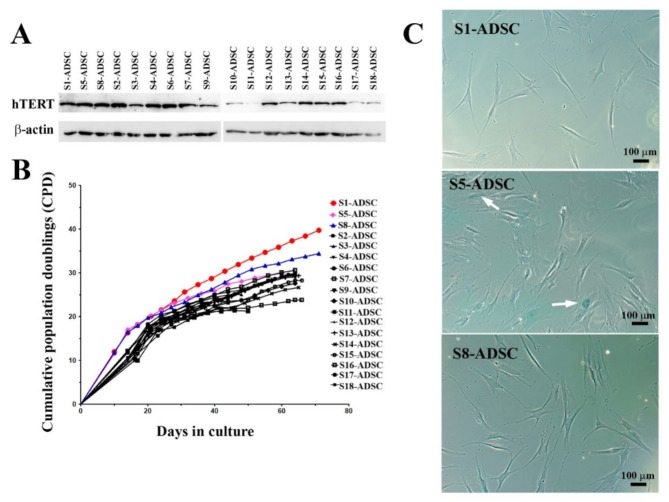
Analysis of hTERT expression, proliferation rates and senescence marker β-galactosidase of hTERT-transduced ADSC-derived sub-populations. (**A**) Expression of hTERT in 18 hTERT-transduced ADSC sub-populations as revealed by Western Blot technique. (**B**) Comparison of proliferation rates of hTERT ADSC-derived sub-populations. Note that two subpopulations (S1 and S8-ADSC) had high proliferation activity, while the rest of the tested cells stopped proliferating after 29–30 population doublings. (**C**) Senescence-associated β-galactosidase assay performed at passage #10 after transduction, showing the lack of enzyme activity in high-proliferative sub-populations S1 and S8-ADSC and a noticeable activity in sub-population S5-ADSC, which stopped proliferating after 30 population doublings (β-galactosidase positive cells are indicated with white arrows).

By serial dilution, 18 sub-populations of hTERT transduced ADSC were obtained. From these, three sub-populations (S1, S5 and S8) stood out from the others for their increased proliferative capacity immediately after cloning, having a population doubling time of 20, 21 and respectively, 20.5 h, compared to the other clones, which performed population doubling in 28 to 40 h. As shown in Figure 3A, the Western blot assay displayed a strong expression of hTERT in most of the sub-populations, with some exceptions (S10, S11, S17, and S18). The comparison of the proliferation rates of all isolated hTERT transduced ADSC sub-populations showed that all clones except for S1 and S8 ceased to grow after approximately 66 days in culture, a figure corresponding to 9 to 30 cumulative population doublings. Clones S1 and S8, however, continued to expand beyond this threshold, reaching 40 and 34 cumulative population doublings, respectively (Figure 3B). Next, the senescence marker β-galactosidase was checked for the 2 highly proliferative clones S1 and S8, but also for S5, which stopped multiplying after 30 population doublings. Note (Figure 3C) the lack of blue stain representing the absence of β-galactosidase marker in S1 and S8 clones, while S5 sub-population had frequent β-galactosidase positive cells, indicating the induction of senescence. Based on these results, we selected S1 and S8 clones to further characterization. 

### 2.4. hTERT-Transduced ADSC Sub-Populations Maintain Their Immunophenotype and Their Multilineage Differentiation Capacity

**Figure 4:**
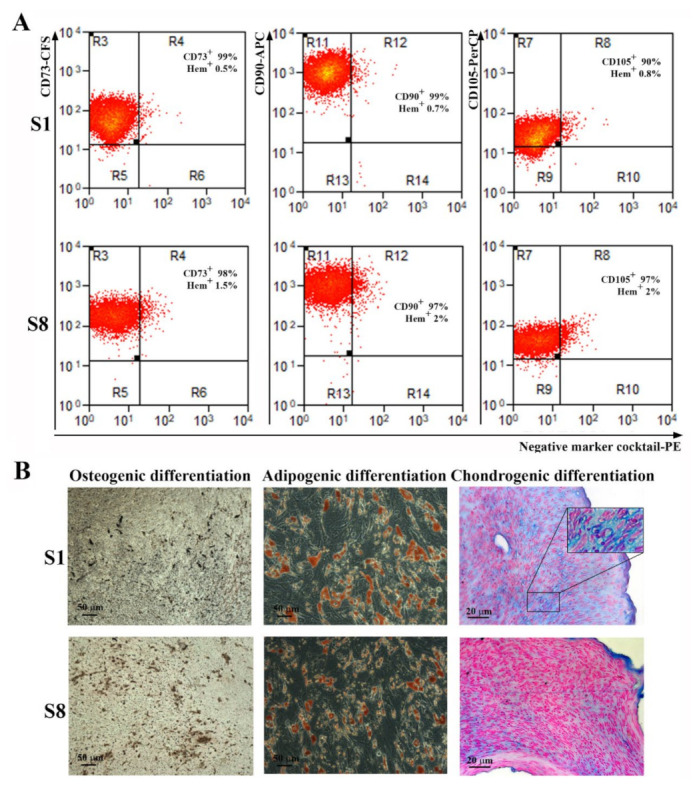
Analysis of surface markers expression and multilineage differentiation capacity of hTERT-transduced ADSC-derived sub-populations. (**A**) Flow cytometry histograms showing the expression of specific surface markers for MSC on sub-populations S1 and S8-ADSC at 5 passages after transduction. It can be noticed that the high expression for CD73, CD90, and CD105 and the absence of hematopoietic markers CD45, CD34, CD11b, CD79A, and HLA-DR were preserved after lentiviral transduction. (**B**) Analysis of the multipotent capacity of hTERT ADSC-derived sub-populations S1 and S8-ADSC by their ability to generate osteocytes (left, von Kossa staining), adipocytes (middle, Oil Red O staining), and chondrocytes (right, Alcian blue staining) when cultured in specific conditions. The insert in the picture of chondrogenic differentiation of S1 sub-population depicts the lacunae-containing chondrocytes.

S1 and S8 sub-population were analyzed to assess whether these cells met the MSC definition requirements. Thus, as shown in Figure 4A, both sub-populations expressed CD73, CD90, and CD105 and were negative for hematopoietic markers. Interestingly, both had a higher expression for CD105 (90% for S1 and 97% for S8) than the parent ADSC or the heterogenous hTERT-transduced ADSC population, which were 86% and 84% CD105 positive, respectively. 

Regarding the multipotent capacity, both S1 and S8 sub-populations maintained their capacity to differentiate into osteoblasts, adipocytes, and chondrocytes when incubated in specific conditions (Figure 4B). However, sub-population S1 demonstrated a remarkable ability for chondrogenic differentiation, since the histological sections of the cells grown in pellets and incubated for three weeks in differentiation conditions displayed not only the presence of Alcian blue-stained acid glycosaminoglycans that are considered markers of chondrogenesis, but also the presence of lacunae, similar to mature cartilage tissue. 

Although clone S5 stopped proliferating and exhibited signs of senescence, we assessed the presence of MSC canonical markers and its trilineage differentiation capacity in order to grasp if there were differences compared to the high-proliferative sub-populations S1 and S8. As seen in Supplemental Figure S3, immunophenotypically and in terms of trilinear differentiation capacity, the S5 clone complied with the accepted MSC characteristics. However, the expression of CD105 was significantly lower (62%), both compared to the S1 and S8 subpopulations (90% and 97%, respectively), and to the initial ADSC and hTERT-induced heterogeneous ADSC population (86% and 84%, respectively). 

Taken together, these data, namely the high proliferative potential, short population doubling time (20 h), the immunophenotype, and the good differentiation potential, prompted us to consider the hTERT-transduced ADSC sub-population S1 as a good candidate to be employed for acellular regenerative therapy. In order to check whether the viral transduction or the long-term culture induced chromosomal aberrations in S1-ADSC, we performed chromosome counting after 36 PD. We found that the transduced cells had a normal chromosome number (Supplementary Figure S4). Next, we evaluated the effect of the conditioned medium (CM) collected from transduced and untransduced ADSC on the main cell types implicated in skin regeneration: keratinocytes, hDF, and EC. 

### 2.5. Factors Released in the Conditioned Medium of hTERT-Transduced ADSC Sub-Population S1 (S1-ADSC) Have a High Capacity to Support Keratinocytes and hDF Viability, Proliferation, and Mobility

**Figure 5:**
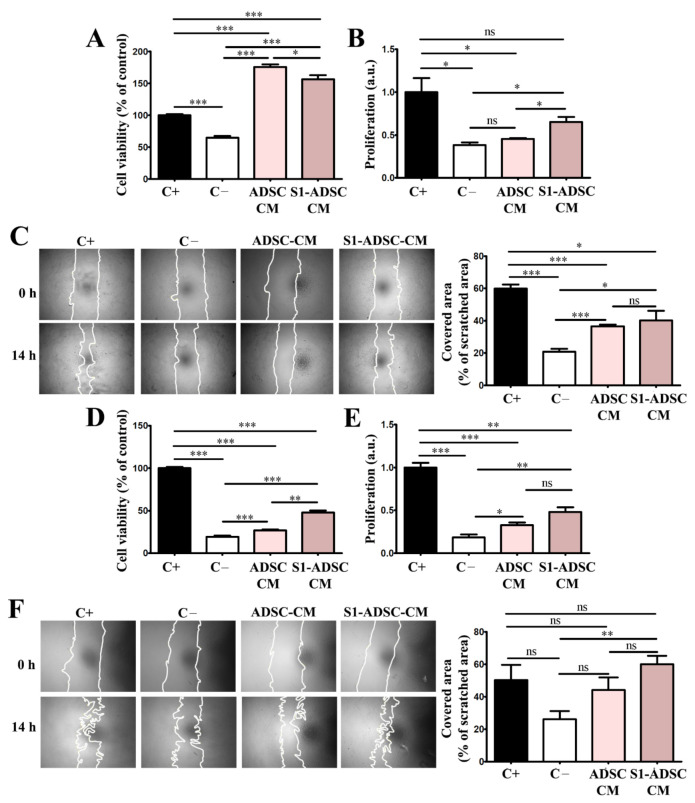
Assessment of the cell viability, proliferation and migration induced by hTERT S1-ADSC-CM on keratinocytes and hDF. (**A**) XTT assay showing the viability of keratinocytes cultured for 5 days in the presence of hTERT S1-ADSC-CM versus ADSC-CM; (**B**) Assessment of the capacity of hTERT S1-ADSC-CM versus ADSC-CM to promote keratinocyte proliferation as revealed by DNA quantification; (**C**) Keratinocyte migration assessed by Scratch test: (left) phase-contrast microscopy showing the scratched area at t = 0 h and 14 h later and (right) the quantification of covered area as a percentage of the initial scratched area. (**D**) XTT assay showing the viability of fibroblasts cultured for 5 days in the presence of hTERT S1-ADSC-CM versus ADSC-CM; (**E**) Assessment of the capacity of hTERT S1-ADSC-CM versus ADSC-CM to promote fibroblast proliferation as shown by DNA quantification; (**F**) Fibroblast migration assessed by Scratch test: (left) phase-contrast microscopy showing the scratched area at t = 0 h and 14 h later and (right) the quantification of covered area as a percentage of the initial scratched area. Data are means ± SD (*n* = 3, *** *p* < 0.001; ** *p* < 0.01; * *p* < 0.05; ns *p* ≥ 0.05, not significant).

As shown in Figure 5A, evaluation by XTT assay revealed that both ADSC-CM and S1-ADSC-CM increased significantly the keratinocytes viability that was 176 ± 9% and 156 ± 14%, respectively, versus 65 ± 7% for the cells incubated in medium without serum (negative control) and 100% for cells incubated in complete medium containing serum (positive control). However, the viability of the keratinocytes exposed to S1-ADSC-CM was slightly lower than that recorded for ADSC-CM. 

As revealed by DNA quantification (Figure 5B), ADSC S1-CM induced the proliferation of keratinocytes by 1.7-fold above the values obtained for the negative control, while ADSC-CM had a statistically insignificant effect on this process. However, the proliferation induced by S1-ADSC-CM did not reach the level of the positive control, which was the complete culture medium. 

Regarding keratinocyte mobility determined by the scratch test, both ADSC-CM and S1-ADSC-CM increased the cells’ capacity to migrate by ~2-fold above the numbers obtained for the negative control but was below the values of the positive control containing serum (Figure 5C). We calculated that ADSC-CM and S1-ADSC-CM covered 36.5 ± 2.5% and 40 ± 13.3%, respectively, of scratched area, versus 20.7 ± 4% for the negative control and 59.8 ± 5.6% for the positive control containing 10% serum. 

Similarly, both ADSC-CM and S1-ADSC-CM sustained hDF viability and proliferation. As shown in Figure 5D, S1-ADSC-CM increased viability by 2.5-fold versus the negative control whereas only a 1.4-fold increase was detected in the case of ADSC-CM. Thus, the effect of S1-ADSC-CM on fibroblast viability was roughly double in comparison to the secretome of untransduced cell population, but was ~50% lower than the positive control (containing serum). Following the same pattern, S1-ADSC-CM increased hDF proliferation by ~2.6-fold versus the negative control. Although the effect of ADSC-CM was slightly lower (1.7-fold increase versus negative control), there was no statistical difference between the two (Figure 5E). However, neither CM induced hDF viability or proliferation at the level of the positive control, suggesting that ADSC-CM does not contain all the factors needed for sustaining these processes. Concerning hDF mobility, only S1-ADSC-CM had a statistically relevant effect (Figure 5F). More exactly, its addition to the cultured cells was associated with two-fold increase in cell migration compared to the negative control (59.9 ± 9.2% versus 26.1 ± 8.5% coverage of scratched area) and was at a similar level with the positive control (50 ± 16.4% coverage of scratched area).

### 2.6. hTERT-Transduced ADSC Sub-Population S1-Derived Conditioned Medium Has Pro-Angiogenic Properties

**Figure 6:**
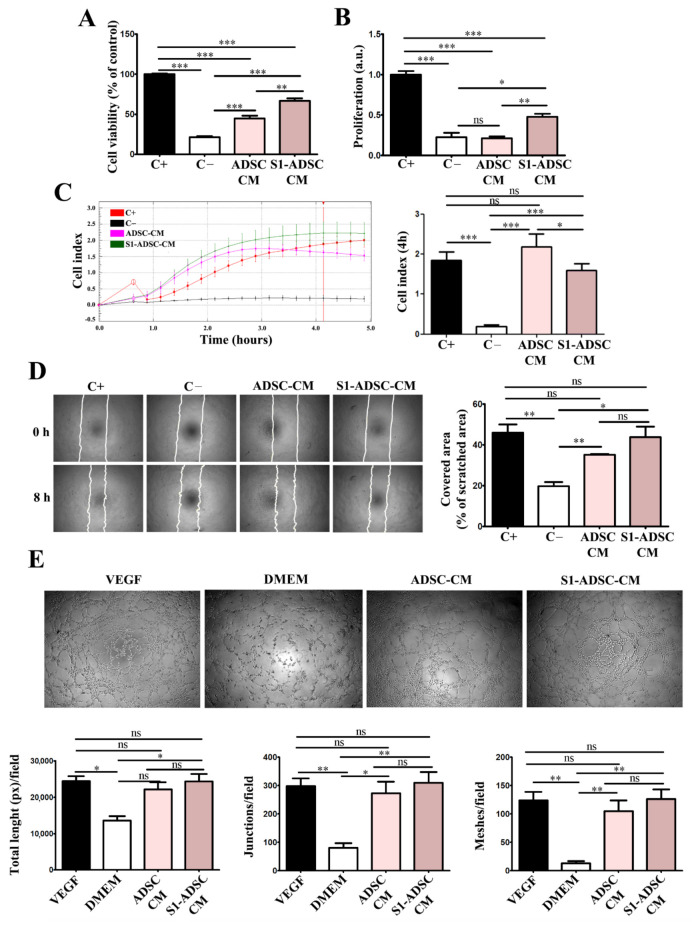
Assessment of pro-angiogenic effect of hTERT S1-ADSC-CM versus ADSC-CM on human EC. (**A**) Viability of EC cultured for 5 days in the presence of hTERT S1-ADSC-CM versus ADSC-CM as revealed by XTT assay; (**B**) Assessment of hTERT S1-ADSC-CM versus ADSC-CM potential to promote EC proliferation as revealed by DNA quantification; (**C**) Time-dependent chemotactic migration of EC. The diagram on the right illustrates the EC migration index at 4 h under the influence of S1-ADSC-CM versus ADSC-CM. (**D**) Evaluation of hTERT S1-ADSC-CM to induce EC migration by Scratch test: (left) phase-contrast microscopy showing the scratched area at t = 0 h and 8 h later and (right) the quantification of covered area as a percentage of the initial scratched area. (**E**) Phase-contrast microscopy showing the angiogenic effect of S1-ADSC-CM versus ADSC-CM on EC using a tube formation assay. The analysis of in vitro tube-like structures by quantification of total tube length (left), junctions (middle), and meshes (right) is presented below. The diagrams show the mean values of one representative experiment performed in triplicates. Data are means ± SD (*n* = 3, *** *p* < 0.001; ** *p* < 0.01; * *p* < 0.05; ns *p* ≥ 0.05, not significant).

To test the angiogenic properties of S1-ADSC-CM, we assessed its effect on the viability, proliferation, chemoattraction, and capacity to form tube-like structures of human EC (Figure 6). We found that S1-ADSC-CM induced a 3-fold increase in EC viability versus the negative serum-free control, while ADSC-CM induced a 2-fold increase only (Figure 6A). Thus, the viability of EC incubated in S1-ADSC-CM was ~66.7 ± 6.6% of that of the positive control, in comparison to 44.6 ± 7.7% for ADSC-CM and 21.3 ± 3.3 for the negative control. Similarly, S1-ADSC-CM induced a 2-fold increase in EC proliferation (in comparison to ADSC-CM, which had no significant (Figure 6B) versus the negative control. 

The time-dependent profile of the chemotactic migration of cultured EC in response to CM was assessed by an xCELLigence assay (Figure 6C). The results showed a strong chemoattractive effect both for S1-ADSC-CM and ADSC-CM. However, although both secretomes had an initial chemoattractive effect stronger than the positive control (serum) at early times (2–3 h), without a statistical significance difference between the two, the cellular index of cells migrating in the presence of S1-ADSC-CM measured at 4 h was about 1.3 times lower in comparison to those incubated with ADSC-CM (2.2 ± 0.3 versus 1.6 ± 0.1, respectively). In addition, the scratch test showed that S1-ADSC-CM had a strong impact on EC migration (Figure 6D). Thus, it induced a coverage of 43.8 ± 8.9% of the scratched area in comparison to 35.2 ± 0.7% for ADSC-CM (not statistically relevant) and 19.7 ± 3.6% for negative control. 

Next, the effect of S1-ADSC-CM on the angiogenic properties of EC was investigated by an in vitro tube formation assay (Figure 6E). The morphometric analysis revealed no statistically significant difference between the S1-ADSC-CM, ADSC-CM, and the positive control consisting in EC incubated in the presence of 50 ng/mL VEGF, a strong pro-angiogenic factor. 

Given the small differences between the effects of the two tested CM, we performed a cytokine array in order to screen the secretion profile of immortalized versus primary ADSC (Supplemental Figure S5). The data showed that the secretome of ADSC consisted of a mixture of pro- and anti-angiogenic factors, similarly to bone marrow-derived MSC [7]. Except for IL-8, a potent pro-angiogenic factor [19], the relative levels of all other pro-angiogenic (angiogenin, IGFBP-2, IGFBP-3, VEGF) and anti-angiogenic (Serpin E1, Serpin F1, TIMP-1, Trombospondin 1) cytokines detected by this technique were lower in S1-ADSC-CM. Pentraxin 3, which is an anti-angiogenic factor, was absent in S1-ADSC-CM. Taken together, these data indicated that S1-ADSC-CM maintained the pro-angiogenic effect on human EC, in spite of the slightly different panel of factors implicated in the regulation of the angiogenic process. 

### 2.7. hTERT-Transduced ADSC Sub-Population S1-Derived Conditioned Medium Stimulate Wound Re-Epithelialization

**Figure 7:**
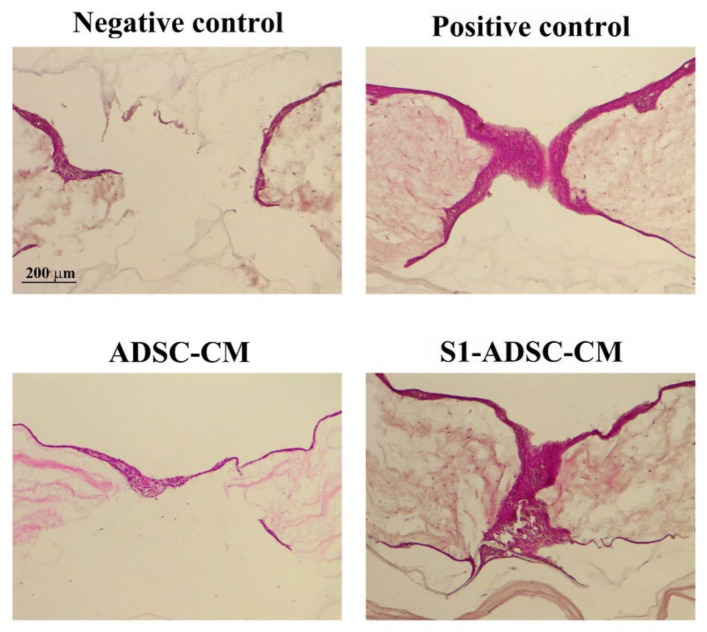
Investigation of S1-ADSC-CM capacity to support wound healing on a human skin organotypic model. Hematoxylin and eosin staining showing the healing of the skin organotypic cultures: after 3 days for the samples incubated with DMEM (negative control), DMEM supplemented with 10% serum (positive control), ADSC-CM, and S1-ADSC-CM. Note that both ADSC-CM and S1-ADSC-CM induced the re-epithelialization of the organotypic culture, similarly with the positive control.

Next, we designed experiments to validate the favorable effects of the condition medium collected from S1-ADSC on keratinocytes, hDF, and EC maintained in 2D culture systems. Thus, we chose to employ a 3D skin organotypic equivalent established in a previous study [18] and to expose the main cell types implicated in skin regeneration (keratinocytes, hDF, and EC) to S1-ADSC-CM. After performing full-thickness wounds using a blunt end needle, the organotypic cultures were exposed to either S1-ADSC-CM or ADSC-CM, or a complete culture medium (positive control) or a basal medium (without serum, negative control). Three days later, a continuous layer of keratinocytes entirely covered the injury site, as shown by hematoxylin and eosin staining (Figure 7). At the same time interval, in the case of the negative control, the organotypic culture presented a still incomplete epidermal layer. These data validated the results obtained with the classical 2D culture system and argued for the noteworthy regenerative effect of S1-ADSC-CM on the epidermal layer of the organotypic culture. 

## 3. Discussion

A problem with using primary cells in regenerative therapy is the cell senescence, which makes their long-term use impossible over several passages. For this reason, it is necessary to obtain continuously isolated cells from patients, which is difficult and could lead to a large variability in the results. A strategy to overcome this challenge is to immortalize primary cells in order to obtain a source of cells that can be used indefinitely, will retain their properties, and generate consistent data. We selected to immortalize ADSC as a source of factors that can be secreted and collected from the conditioned medium. The expression of the hTERT gene (telomerase) was chosen, because it has the role of maintaining the size of the telomeric ends of the chromosomes, thus repressing the replicative senescence. It is also known that cells immortalized with hTERT have a normal karyotype, maintain the phenotypic characteristics of primary cells and control of the cell cycle, respond normally to serum and mitogens, are not malignant, exhibit contact inhibition, and are dependent on anchoring to the substrate [20]. Other groups showed the possibility of obtaining immortalized MSC lines using only hTERT [13,21,22]. In this study, we aimed to obtain an ADSC population capable of lasting self-renewal and able to secrete factors with therapeutic properties for skin wound healing. For this purpose, we used lentiviral transduction in order to induce the expression of hTERT in a primary culture of ADSC. As described above, following selection with hygromycin, a heterogenous cell population was obtained, which was assessed for hTERT expression and for stem cell characteristics. Thus, although hTERT was expressed in the transduced cells, while the band in the initial cells was barely visible, the chondrogenic differentiation was impaired. Due to the heterogenous nature of this population, we assumed that the paracrine properties of such population could be modified with passages, which does not allow the standardization of culture conditions for obtaining the secretome neither for research purposes, nor for clinical applications. Thus, we proceeded to single cell cloning by using a serial dilution technique, which resulted in obtaining 18 clones. From these, 3 sub-populations, notably S1-ADSC, S5-ADSC, and S8-ADSC, were highlighted due to their high proliferation capacity shortly after the cloning process. However, only sub-populations S1 and S8 were able to proliferate more than 30 population doublings, while all the other clones ceased to multiply and exhibited the presence of β-galactosidase, a senescence marker, including the initially highly proliferative S5-ADSC clone. Interestingly, as shown in the supplemental data, although the differentiation potential of the S5 clone was not affected by the transduction, it had a lower expression of CD105 (about 60%) when compared to the initial population or to the S1-ADSC and S8-ADSC sub-populations (80% and over 90%, respectively). There are controversial data in the literature concerning the proliferation capacity of CD105 negative ADSC: some studies reported no difference between the proliferation ability of CD105-positive and CD105-negative ADSC [23,24], while another paper showed increased cell proliferation and colony formation for CD105-positive as compared to the negative control [25]. The connection between CD105 expression and proliferation capacity cannot be determined from the available data and needs further investigation.

The characterization of S1- and S8-ADSC-derived sub-populations showed that both met the requirements of the International Society for Cellular Therapy [18]. Still, S1-ADSC had a superior capacity to differentiate toward chondrocytes as compared not only to the other sub-populations, but also with the primary ADSC. In this case, the greater differentiation was revealed by the presence of numerous chondrocytes within lacunae (not found in any other conditions) resembling the fully differentiated cartilage. Consequently, we chose the transduced S1-ADSC sub-population for further assessment of the regenerative properties in wound healing. We tested the direct effect of the secretome collected from S1-ADSC on the main cell types involved in the wound healing process in a 2D culture system, and its regenerative capacity on a 3D skin organotypic culture.

First, we report here the impact of S1-ADSC-CM on the proliferation and migration of keratinocytes, hDF, EC, and on angiogenesis, which are processes occurring during the proliferative phase of wound healing [26]. Our data showed that both ADSC-CM and S1-ADSC-CM sustained these processes, however, the latter had a slightly superior effect, although not always statistically relevant. An interesting observation came from the viability measurement by XTT assay and proliferation assessment by DNA quantification. Although both techniques are usually used to assess cell proliferation, in our experiments the two methods did not provide identical results. In the case of the proliferation of keratinocytes and EC, the effect of ADSC-CM was stronger in the XTT assay than by DNA quantification. Thus, ADSC-CM did not induce cell proliferation as seen by DNA quantification, while it strongly induced viability as shown by the XTT assay. This could be explained by the difference in the principles of the two methods. Thus, although an XTT assay can be used for determining the proliferation of a cell culture, it is based on their metabolic activity, which is the extracellular reduction of XTT by NADH produced in the mitochondria via trans-plasma membrane electron transport and an electron mediator [27], while the quantification of DNA is a direct measure of DNA synthesis. Our results could indicate that ADSC-CM, as well as S1-ADSC-CM, induce an increased metabolic activity in keratinocytes and EC. It was previously shown that ADSC-CM was able to facilitate neuroprotection by regulating energy metabolism (including the increase in NADH) [28]. Thus, we presume that, at least partially, the effect of CM on these two cell types could be mediated by the regulation of energy metabolism. This pattern was not observed for hDF.

In addition, both ADSC-CM and S1-ADSC-CM had a positive impact on angiogenesis, which is a key process, because it provides the foundation for tissue survival and recovery in the wound [29]. Our data showed that only S1-ADSC-CM promoted EC proliferation, as revealed by DNA quantification. Moreover, it sustained EC chemotaxis, migration, and formation of tube-like structures on Geltrex. We also showed that, although the composition of immortalized cell-derived secretome was slightly altered, the overall pro-angiogenic effect was preserved. This may be explained by the fact that the level of IL-8, a potent pro-angiogenic cytokine, was maintained, even though the VEGF level was lower in S1-ADSC-CM. Moreover, in a previous study we showed that the function of VEGF can be substituted by the presence of SDF-1α [30]. However, the latter was not included in the cytokine array that we used and, in order to affirm that this mechanism is implicated in the preservation of the pro-angiogenic activity of S1-ADSC-CM, further studies are required.

Our results corroborate well with a recent study on an immortalized ADSC line with beneficial properties on EC and fibroblasts cultured in a 2D classical system [22]. Our experiments bring additional relevant data, especially those using the in vitro 3D full-thickness wound model of a human skin organotypic culture. As is generally accepted, the 3D model, by allowing complex interaction between various cell types, provides a good alternative for pre-clinical drug testing, which is otherwise restricted by ethical issues and differences between human and animal biology. We have shown here that the secretome derived from transduced S1-ADSC induced a complete re-epithelialization of the full-thickness wound on the 3D human skin organotypic culture model. The results were similar to those obtained with the secretome collected from untransduced (native) cells validating that the transduction process did not affect the main properties of ADSC. However, this model has limitations regarding the absence of other cell types involved in the wound healing process in vivo, such as immune cells [31]. It was previously shown that ADSC-CM induces an anti-inflammatory macrophage response by promoting an M2 macrophage phenotype [32] via ADSC-released exosomes [33,34]. We assume that this mechanism could also be implicated in skin wound healing sustained by S1-ADSC-CM, but further tests are needed. Additionally, the capacity of fibroblasts to secrete extracellular matrix proteins (collagens, fibronectin, elastin) could be modulated by S1-ADSC-CM and needs further testing.

In conclusion, we selected an immortalized S1-ADSC line that maintained the key characteristics of the primary cells. Moreover, these cells released in the secretome factors that have an effect similar and sometimes slightly superior over the original non-transduced cells and on the main cell types involved in wound healing, and sustained the re-epithelialization process on a human skin organotypic wound model. This is a good alternative to the whole-cell therapy approach: the use of cultured immortalized selected cell lines as a source of growth factors and cytokines that they release in a conditioned medium that have the capacity to enhance the proliferation of skin-derived cells and the therapy of wound healing.

## 4. Materials and Methods

### 4.1. Cell Culture

All the procedures involving human samples were approved by the Institutional Ethical Committee (180/27.09.2018), in accordance with the recent version of the Helsinki declaration of the World Medical Association (Ethical Principles for Medical Research Involving Human Subjects, October 2008). Each donor signed an informed written consent form for collecting and using cutaneous and adipose tissue.

ADSC were isolated from abdominal subcutaneous adipose tissue samples harvested during abdominoplasty of a healthy patient. Briefly, the connective tissue and blood vessels were removed, and the adipose sample were minced and digested in type I collagenase 0.5 mg/mL (Sigma Aldrich, St. Louis, MO, USA) with stirring for 30–40 min/37 °C. After adding an equal volume of low glucose (1 g/L) Dulbecco’s Modified Eagle Medium (DMEM) from Sigma Aldrich, St. Louis, MO, USA, supplemented with 10% (*v*/*v*) fetal bovine serum (FBS) from Gibco BRL, Gaithersburg, MD, USA, and 100 IU/mL penicillin, 100 µg/mL streptomycin, 50 µg/mL neomycin (Sigma Aldrich, St. Louis, MO, USA), the digested tissue was centrifuged at 200 g for 10 min. The pellet was suspended in complete medium and filtered through a 100 µm sieve (BD Falcon Cell Strainer, Franklin Lakes, NJ, USA). After centrifugation (200 g, 10 min), the cells were resuspended in low-glucose DMEM supplemented with 1% non-essential amino acids (Sigma Aldrich, St. Louis, MO, USA), 10% of FBS, and 100 IU/mL penicillin, 100 µg/mL streptomycin, 50 µg/mL neomycin (Sigma Aldrich, St. Louis, MO, USA) and cultured in standard conditions (37 °C in 5% CO_2_ atmosphere). The medium was changed at 24 h after isolation, and then every 2 days. The cells were passaged at ~70% confluency.

DF were isolated from human skin samples from plastic surgery procedures, with informed consent, as previously described [7] and grown in low glucose DMEM supplemented with 1% (*v*/*v*) non-essential amino acids (Sigma Aldrich, St. Louis, MO, USA), 10% FBS, and 100 IU/mL penicillin, 100 µg/mL streptomycin, 50 µg/mL neomycin (Sigma Aldrich, St. Louis, MO, USA), at 37 °C and 5% CO_2_.

Human keratinocyte cell line HaCaT (CLS GmbH, Eppelheim, Germany) and human endothelial cell line EA.hy926 (ATCC, CRL-2922, Manassas, VA, USA) were employed according to the manufacturer’s instructions. Briefly, the cells were cultivated in high (4.5 g/L) and low glucose DMEM (Sigma Aldrich, St. Louis, MO, USA), respectively, with 1% non-essential amino acids (Sigma Aldrich, St. Louis, MO, USA), 10% FBS, and 100 IU/mL penicillin, 100 µg/mL streptomycin, 50 µg/mL neomycin (Sigma Aldrich, St. Louis, MO, USA), at 37 °C, 5% CO_2_.

The virus packaging AD-293 cells (Agilent Technologies, Santa Clara, CA, USA), a transformed human embryonic kidney cell line, were grown in high-glucose DMEM supplemented with 10% heat-inactivated FBS and 100 IU/mL penicillin, 100 µg/mL streptomycin, 50 µg/mL neomycin (Sigma Aldrich, St. Louis, MO, USA), in a 5% CO_2_ containing humidified atmosphere at 37 °C.

### 4.2. Cell Phenotyping

ADSC were detached from the plate using Accutase solution (Sigma Aldrich, St. Louis, MO, USA, A6964) and incubated with the following antibodies that fulfill the minimal criteria for the definition of human MSC: anti-CD73 conjugated with carboxyfluorescein (CFS), CD90–allophycocyanin (APC) conjugated antibody, CD105–Peridinin-Chlorophyll-Protein (PerCP) conjugated antibody, the antibodies included in the Negative Marker Cocktail (anti-CD45, anti-CD34, anti-CD11b, and anti-HLA-DR) coupled with PE (phycoerythrin), and isotype controls, according to the manufacturer’s protocol, 10 µL each in 100 µL cell suspension containing 10^5^ cells (Human Mesenchymal Stem Cell Verification Flow Kit, R&D Systems, Minneapolis, MN, USA). The samples were evaluated using a Beckman Coulter 3 laser Gallios cytometer and the data analysis was performed using Summit software v4.3 (Cytomation, Inc, Fort Collins, CO, USA).

### 4.3. Multilineage Differentiation Assays

The multilineage potential of ADSC was tested by searching their capacity to differentiate into adipogenic, osteogenic, and chondrogenic lineages. Briefly, for adipogenic differentiation, confluent cells were incubated in adipogenic differentiation medium (low-glucose DMEM with 10% FBS, 10^−6^ M dexamethasone, 0.5 mM IBMX (3-isobutyl-1-methylxanthine), 1 µg/mL insulin, and 1µM rosiglitasone, (all from Sigma Aldrich, St. Louis, MO, USA) for 21 days. The cells were then stained with Oil Red O (Sigma-Aldrich, Saint Louis, MO, USA) to determine the presence of intracellular lipid droplets. For osteogenic differentiation, confluent cells were incubated in osteogenic differentiation medium (low-glucose DMEM with 10% FBS, 10^−7^ M dexamethasone, 10 mM β-glycerophosphate and 0.2 mM ascorbic acid, all from Sigma Aldrich, St. Louis, MO, USA) for 21 days. The calcium deposits were highlighted by von Kossa staining. For chondrogenic differentiation, 2.5 × 10^5^ cells were resuspended in 500 µL chondrogenic differentiation medium (high glucose-DMEM with 10 ng/mL TGF-β3, 10^−7^ M dexamethasone, 50 ug/mL ascorbate-2-phosphate, 1% insulin-transferrin-sodium selenite & linoleic-BSA (ITS + 1), all from Sigma Aldrich, Saint Louis, MO, USA), in 15 mL Falcon tubes. The tubes were centrifuged at 400 g, for 5 min before being moved into the CO_2_ incubator at 37 °C for 21 days. Gentle medium changing was carried out three times per week taking care to not disperse the cell pellet. The pellets were embedded in paraffin, sectioned at 5 μm, and stained with Alcian Blue (Sigma-Aldrich, Saint Louis, MO, USA) to highlight the acid mucopolysaccharides.

### 4.4. Trasduction of ADSC with hTERT

For the transduction of ADSC, a third generation of a lentiviral packaging system was employed [35]. The plasmids used were the packaging plasmids pMD2.G (Addgene plasmid #12259) and pMDLg_pRRE (Addgene plasmid #12251), the regulatory plasmid pRSV-Rev (Addgene plasmid #12253), a gift from Didier Trono, and the hTERT codifying plasmid (pLV-hTERT-IRES-hygro), a gift from Tobias Meyer (Addgene plasmid #85140), Addgene, Watertown, MA USA. The lentivirus packaging was carried out using AD293 cells (transformed human embryonic kidney cell line, Agilent, Santa Clara, CA, USA). Briefly, using Lipofectamine 3000 Transfection Reagent (Invitrogen, Bleiswijk, The Netherlands), the four plasmids were simultaneously introduced into AD293 cells, in the pRSV-Rev: PMDLg_pRRE: pMD2.G: PL-hTERT-IRES-hygro report of 1:2:1:4, following the manufacturer’s instructions. After 3 days, the medium containing the lentiviral particles was collected, filtered through 0.45 µm pore filters and added over pre-confluent ADSC, in various dilutions of viral medium in the presence of 8 μg/mL Polybrene (Sigma-Aldrich, Saint Louis, MO, USA). A well was kept as a control to verify the effectiveness of the antibiotic selection. After 24 h of incubation, the medium was removed and fresh medium (DMEM with 10% FSB and 100 IU/mL penicillin, 100 µg/mL streptomycin, 50 µg/mL neomycin, all from Sigma-Aldrich, Saint Louis, MO, USA) supplemented with 200 mg/mL hygromycin B (Sigma-Aldrich, Saint Louis, MO, USA) was added.

### 4.5. Cell Cloning

For clonal selection, one single cell per well was plated in a 96-well plate and only wells with one colony per well were considered monoclonal. After identification of the wells with a single colony, the colony was checked daily until they reached sub-confluence. All subpopulations were grown in the presence of 100 mg/mL Hygromycin B (Sigma-Aldrich, Saint Louis, MO, USA). Cells were tripsinized, counted for determination of cell doublings, and plated in a 24-well plate and next in a 6-wells plate dish at a density of 10,000 cells/cm^2^.

### 4.6. Western Blot Assay

Cells were lysed using Laemmli Sample Buffer (Sigma Aldrich, St. Louis, MO, USA) supplemented with a protease inhibitor cocktail (Sigma Aldrich, St. Louis, MO, USA). Ten µg/mL total proteins were separated on 10% polyacrylamide gel (SDS-PAGE) and transferred on a PVDF membrane (Merck Millipore, Burlington, MA, USA). After blocking-in in 5% non-fat powdered milk (Sigma Aldrich, St. Louis, MO, USA) for 1 h at room temperature, the membranes were incubated with primary antibodies against hTERT (1:250 dilution, 19HCLC, Thermo Fisher Scientific, Waltham, MA, USA) and β-actin (1:1000 dilution, A5441, Sigma Aldrich, St. Louis, MO, USA) overnight, at 4 °C. The next day, the membranes were washed and incubated with the corresponding HRP-conjugated secondary α-mouse and α-rabbit antibodies (1:10,000 dilution each, Thermo Fisher Scientific, Waltham, MA, USA) at room temperature for 1 h. The data were collected using a Luminescent Image analyzer LAS-3000 (FUJIFILM, Tokyo, Japan) after incubation in Immobilon Forte HRP Substrate (Merck Millipore, Burlington, MA, USA). The densitometric analysis was performed using the Scion Image software (Scion Corp., Frederick, MD, USA).

### 4.7. Cell Proliferation Kinetics

ADSC were plated at density of 1 × 10^5^ cells/cm^2^, and at ~70–80% confluence were trypsinized and replated. The same procedure was repeated for multiple passages with cell counting at each passage. Long-term cell growth was checked by evaluation of the cumulative population doubling (CPD) and population doubling time (PDT). The population doublings (PD) were calculated according to standard formula [36]: ln 2 × ln (Nf/N0), and PDT was considered T/PD, where Nf–cell number at the end of a passage, N0–seeded cell number, and T–culture time (hours) of the experimental period (from N0 to Nf). CPD represents the sum of the population doublings and was plotted against time in culture (days) for determination of growth kinetics.

### 4.8. SA-β-Galactosidase Assay

ADSC were seeded at a density of 2.5 × 10^4^ cells/well in a 96-well plate. After 48 h, senescence-associated β-galactosidase (SA-β-Gal) expression was detected using Senescence Detection Kit (ab28364, Abcam, Cambridge, UK,), according to the manufacturer’s instructions. The images of the blue staining specific to senescent cells were acquired with a Zeiss Axiovert Observer D1 microscope (Zeiss, Oberkochen, Germany).

### 4.9. Collection of ADSC Conditioned Medium

hTERT-transduced ADSC (17th passages after transfection) and parental ADSC were seeded at a density of 1 × 10^4^ cells/cm^2^ and cultured to achieve 70–80% confluence. Then, the cells were washed with PBS (phosphate buffered saline) and incubated with DMEM for 24 h. Conditioned medium was collected, centrifuged (2000 g, 25 min), and then concentrated using Amicon Ultra Spin Columns 10 k (Merck, Kenilworth, NJ, USA) according to the manufacturer’s protocol, and stored at −80 °C. In the cell culture experiments, concentrated CM was added as a 10% supplement in serum-free medium.

### 4.10. Cell Viability Assessment

Cells were seeded in complete medium on 96-well plates (1 × 10^4^ cells/cm^2^). After 24 h, the medium was replaced with serum-free low-glucose DMEM (negative control), low-glucose DMEM with 10% serum (positive control), and low-glucose DMEM with 10% CM. After 5 days, the cell proliferation was evaluated by an XTT assay (Thermo Fisher Scientific, Waltham, MA, USA). The data are given as percentage of the positive control.

### 4.11. Cell Proliferation Assay

Cell proliferation was assessed by fluorescent staining of the DNA. The cells (1 × 10^4^ cells/cm^2^) were seeded onto 24-well plates and at confluence were incubated with CM (ADSC-CM and S1-ADSC-CM) for 5 days. After a brief wash with PBS, the cells were lysed by liquid nitrogen. Cells were incubated for 1 h with the nucleic acid dye Hoechst 33258 (Sigma-Aldrich, St. Louis, MO, USA). The fluorescence, which is proportional to the number of cells, was measured in black flat-bottom plates using a TECAN fluorescence plate reader (TECAN, Männedorf, Switzerland) at 350 nm excitation and 460 nm emission wavelengths.

### 4.12. Cell Migration Assay (Scratch Test)

Keratinocytes, fibroblasts, and EC were grown to confluence in 96-well plates. The monolayer was scratched transversely using a 200-μL pipette tip, washed with PBS and incubated in complete medium (positive control), serum-free DMEM (negative control), and CM (ADSC-CM and S1-ADSC-CM). Pictures were taken at 0 h and at 8 h for EC and 14 h for keratinocytes and fibroblasts with a Zeiss Axiovert Observer D1 microscope (Zeiss, Oberkochen, Germany). The migration of the cells was quantified using the ImageJ software (NIH, Bethesda, MD, USA).

### 4.13. Chemotaxis Assay

The chemotactic properties of the secretome (CM) harvested from hTERT-transduced ADSC single-cell derived clones were evaluated using the xCELLigence RTCA system (Acea Biosciences, Inc, San Diego, CA, USA). In brief, EC (1 × 10^4^ cells/100 µL medium) were added onto the upper well of the CIM-plate 16. The tested media (complete medium—positive control, serum-free DMEM—negative control, and CM) were added to the lower chamber of the CIM-plate and the cell migration through the membrane was checked in real-time for 24 h.

### 4.14. Tube Formation Assay

EC (4.5 × 10^4^ cells/cm^2^) were seeded on 96-well plates coated with 50 µL of Geltrex, a growth factor-reduced basement membrane matrix (Thermo Fisher Scientific, Waltham, MA, USA) in either DMEM supplemented with 50 ng/mL VEGF (positive control), DMEM without FBS (negative control), or with CM (ADSC-CM and S1-ADSC-CM). Images were taken after 8 h using a Zeiss Axiovert microscope (Zeiss, Oberkochen, Germany) and analyzed with ImageJ software (NIH, Bethesda, MD, USA).

### 4.15. Human Skin Organotypic Culture

The production of human skin organotypic cultures (OTC) has been previously established [37]. In short, the dermal equivalents were obtained by mixing fetal bovine serum containing 3.75 × 10^5^/mL hDF with 10× Hank’s buffered saline (Sigma Aldrich, St. Louis, MO, USA) and bovine type I collagen (Thermo Fisher Scientific, Waltham, MA, USA) in a proportion of 1:1:8 (volumes). The gel was cast on 24-well polycarbonate inserts (Merck Millipore, Burlington, MA, USA) and complete culture medium was added in both compartments for 24 h. Next, 1 × 10^5^ keratinocytes/insert (HaCaT cell line) were seeded on top of the collagen layer. After 4 days, the 3D culture was raised to the air–liquid interface, and the medium was supplemented with 2 ng/mL TGFα (Thermo Fisher Scientific, Waltham, MA, USA), 100 ng/mL GM-CSF (Thermo Fisher Scientific, Waltham, MA, USA), and ascorbic acid (Sigma Aldrich, St. Louis, MO, USA) to a final concentration of 50 mg/L. After another 3 days, a blunt needle was used to perform a full-thickness lesion. The wounded constructs were transferred on top of another collagen gel containing fibroblasts and incubated in complete medium (positive control), serum-free DMEM (negative control), and CM (ADSC-CM and S1-ADSC-CM). The skin equivalents were fixed in 4% PFA at day 0 and day 3 post-wound and processed for cryosectioning. The cryosections were stained with hematoxylin and eosin and the slides were examined using a Leica DM750 microscope (Leica, Wetzlar, Germany).

### 4.16. Statistical Analysis

Data were analyzed using the software GraphPad Prism 5.0 (GraphPad, San Diego, CA, USA) and are presented as mean ± standard deviation (SD) from at least three independent experiments, measured in triplicates. Statistical significance was determined using Student’s *t*-test (*** *p* < 0.001; ** *p* < 0.01; * *p* < 0.05; ns *p* ≥ 0.05).

## Data Availability

Data available on request from the authors.

## Supplementary Materials

The supporting information can be downloaded at: https://www.mdpi.com/article/10.3390/ijms23168925/s1.
