# Supplementary material for: Generation of an Immortalized Human Adipose-Derived Mesenchymal Stromal Cell Line Suitable for Wound Healing Therapy
Source: Int J Mol Sci. 2022 Aug 10;23(16):8925. doi: 10.3390/ijms23168925 (PMC9408591; doi:10.3390/ijms23168925)
Supplement: Supplementary file 1 [file ijms-23-08925-s001.zip › ijms-1819667-supplementary.pdf]

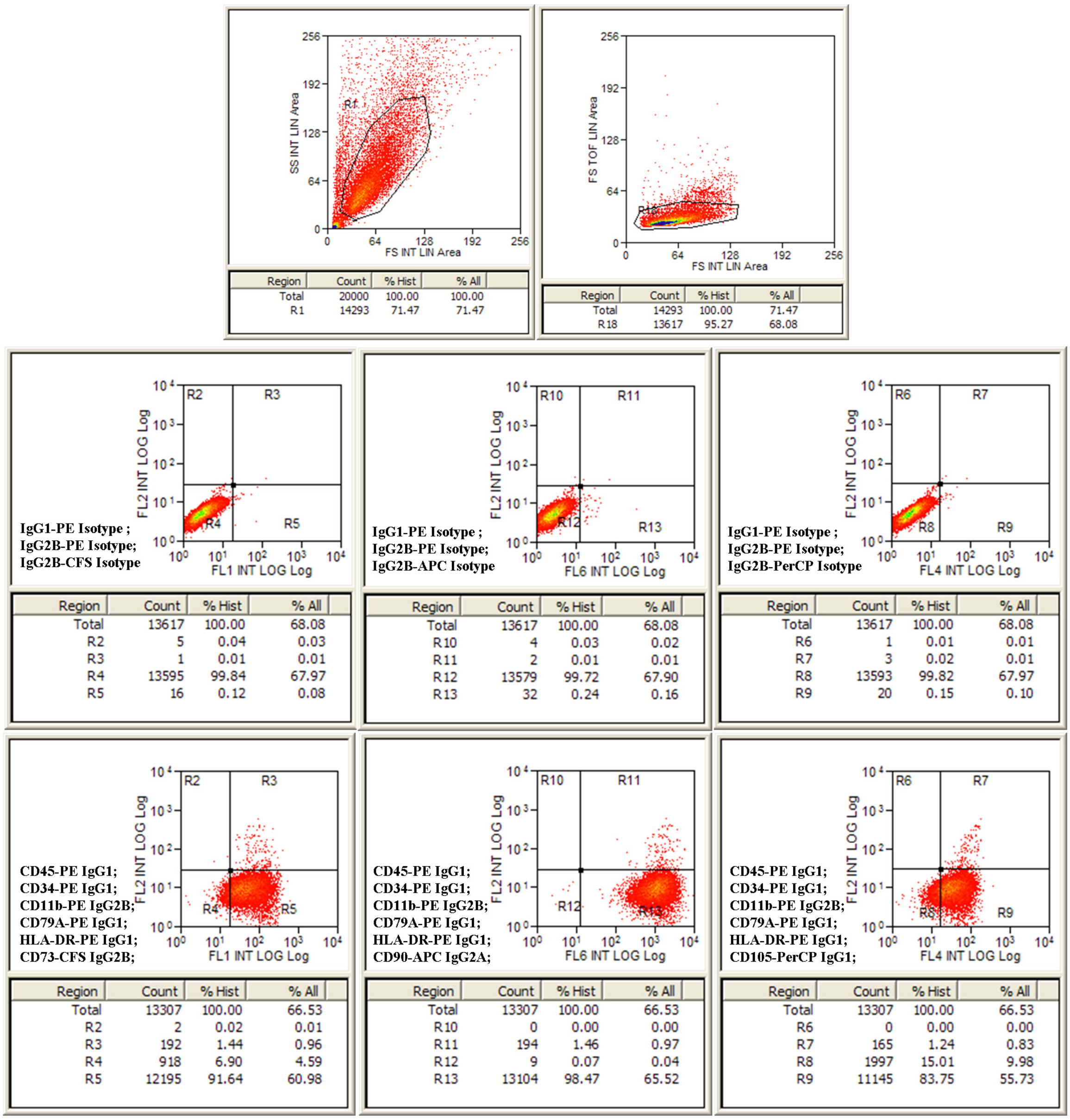

Figure S1: Gating strategy used for flow cytometry analysis and negative controls for each antibody.

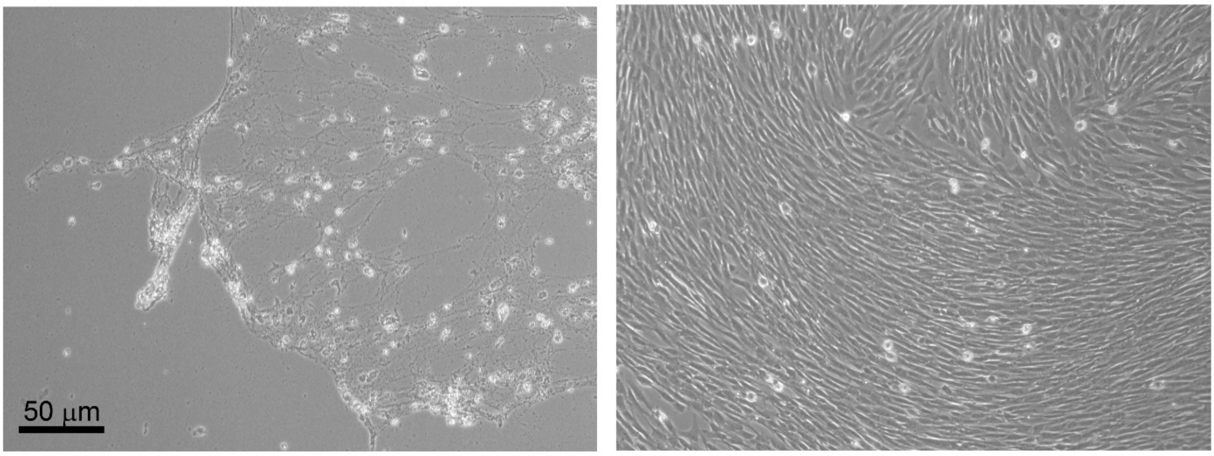

Figure S2. Phase contrast image of hTERT transduced ADSC after three weeks of hygromycin selection. The left image shows the dead un-transduced cells after hygromycin incubation, and right panel shows the fibroblast-like morphology of selected cells after hTERT lentiviral transduction of ADSC.

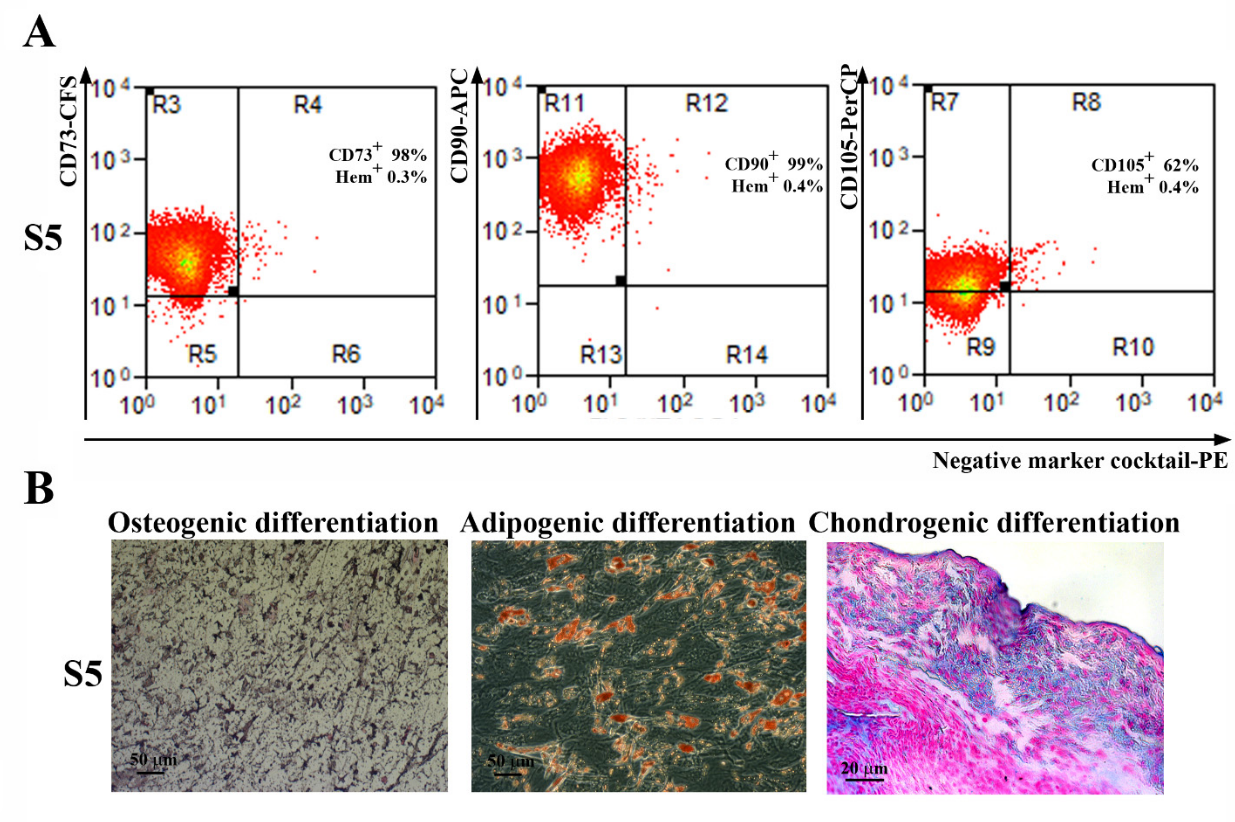

Figure S3. Surface markers expression and multilineage differentiation capacity of S5-ADSC sub-population. (A) Flow cytometry histograms showing the expression of specific surface markers for MSC on sub-population S5, 11passages after transduction. It can be noticed that CD105 expression was only 60%. (B) Analysis of the multipotent capacity of hTERT ADSC derived sub-population S5 by their ability to generate osteocytes (left, von Kossa staining), adipocytes (middle, Oil Red O staining), and chondrocytes (right, Alcian blue staining) when cultured in specific conditions.

Chromosomes counting

To obtain chromosomes at the mitotic (metaphase) stage, cells in the exponential growth phase (80% confluency) were treated overnight with 0.01 µg/mL Colchicine (Sigma Aldrich, St. Louis, MO, USA). Then, the cells were gently washed with HBSS Buffer, trypsinized and washed with PBS, according to standard procedures. After centrifugation, cells were resuspended in 0.075 M KCl and incubated for 10 min at 37°C. Following the hypotonic treatment, the samples were fixed with fresh Carnoy's Fixative (3:1 ratio of methanol: acetic acid). The cells were spread onto slides by dropping of cell suspension and the metaphase spreads were mounted with Fluoroshield with DAPI (Sigma Aldrich, St. Louis, MO, USA) and photographed with Zeiss Observer D1 microscope (Zeiss, Oberkochen, Germany). Fifteen metaphase images per sample were used for chromosome counting.

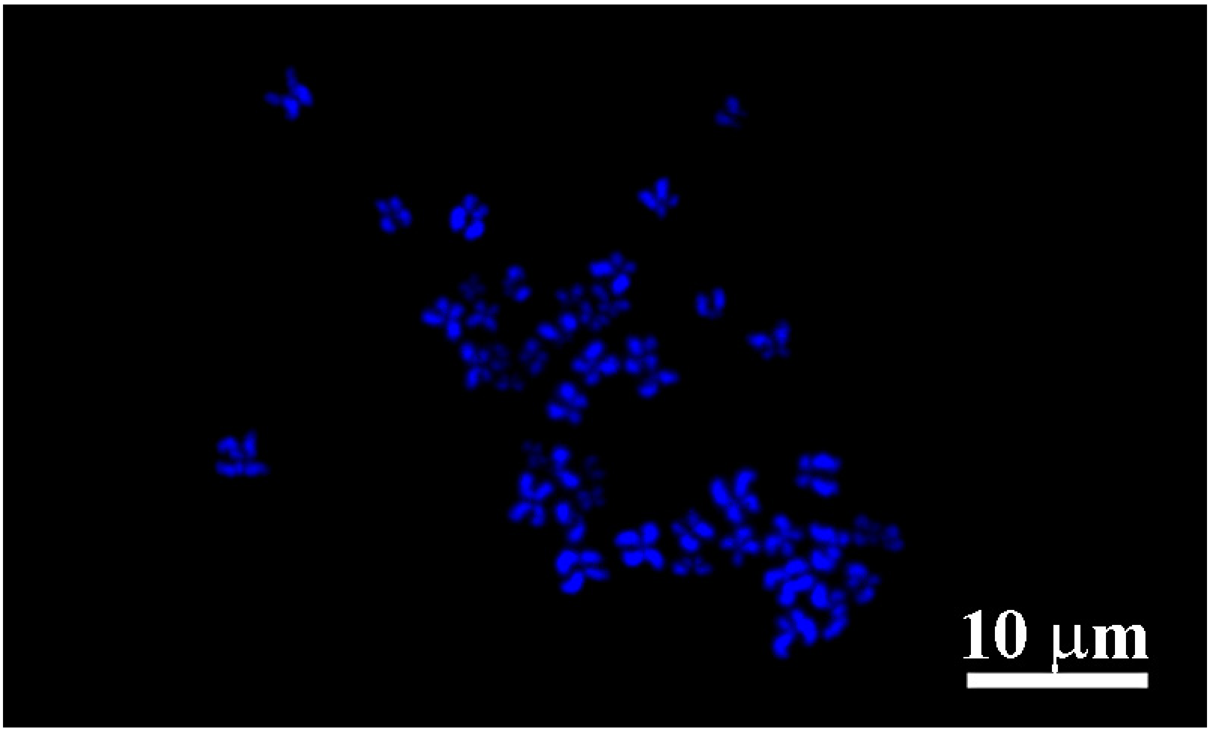

Figure S4: Visualization of metaphasic chromosomes with DAPI staining

Cytokine array

The relative levels of angiogenesis-related proteins synthesized in S1-ADSC-CM vs ADSC-CM were analyzed using the ARY007 Proteome Profiler Angiogenesis Array Kit (R&D Biosystems, Minneapolis, MN, USA), according to the protocol of the manufacturer. Briefly, the array membranes were blotted and incubated overnight with the CM (harvested from 10^6 cells) and with the detection antibody cocktail. Then, the membranes were submerged in wash buffer to remove non-attached proteins and incubated with horseradish peroxidase-conjugated streptavidin antibody. After exposure to detection reagent, the membranes were then simultaneous imaged using a Luminescent Image analyzer LAS-3000 (FUJIFILM, Tokyo, Japan).

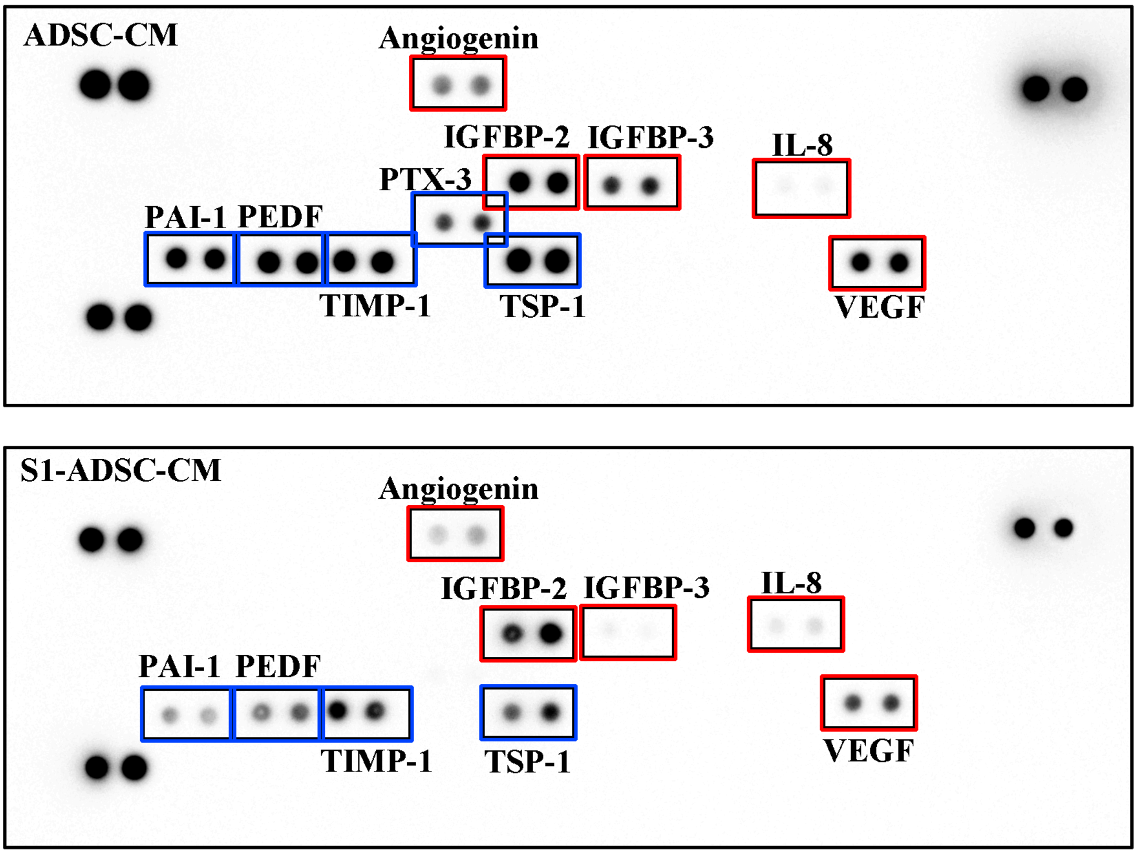

Figure S5: Cytokine array for determination of the relative levels of angiogenesis-related proteins: IGFBP-2 - Insulin-Like Growth Factor Binding Protein-2, IGFBP-3 - Insulin-Like Growth Factor Binding Protein-3, IL-8 -Interleukin -8, PAI-1 – Serpin E1, PEDF – Serpin F1, TIMP-1 - Tissue Inhibitor Matrix Metalloproteinase 1, TSP-1 - Thrombospondin 1, VEGF - Vascular endothelial growth factor.
